# Seropositivity of *Leptospira* spp. Antibodies among Febrile Patients Attending Outpatient Clinics in Mwanza, Tanzania: Should It Be Included in Routine Diagnosis?

**DOI:** 10.3390/tropicalmed7080173

**Published:** 2022-08-09

**Authors:** Mariam M. Mirambo, Vitus Silago, Betrand Msemwa, Helmut Nyawale, Mlekwa G. Mgomi, Julius M. Madeu, William S. Nasson, Gabriel Emmanuel, John Moses, Namanya Basinda, Ginethon Mhamphi, Subira S. Mwakabumbe, Elifuraha B. Mngumi, Khadija S. Majid, Lucas Matemba, Georgies Mgode, Stephen E. Mshana

**Affiliations:** 1 Department of Microbiology and Immunology, Catholic University of Health and Allied Sciences, Bugando, Mwanza P.O. Box 1464, Tanzania; 2 Department of Medical Laboratory Sciences, Institute of Allied Health Sciences, Catholic University of Health and Allied Sciences, Bugando, Mwanza P.O. Box 1464, Tanzania; 3 Department of Community Medicine, School of Public Health, Catholic University of Health and Allied Sciences, Bugando, Mwanza P.O. Box 1464, Tanzania; 4 SUA Pest Management Centre (SPMC), Sokoine University of Agriculture, Morogoro P.O. Box 3110, Tanzania; 5 Veterinary Investigation Centre (VIC), Lake Zone, Ministry of Livestock and Fisheries, Mwanza P.O. Box 129, Tanzania; 6 Department of Veterinary Anatomy and Pathology, School of Biomedical Sciences, Sokoine University of Agriculture, Morogoro P.O. Box 3016, Tanzania; 7 Department of Veterinary Medicine and Public Health, Sokoine University of Agriculture, Morogoro P.O. Box 3021, Tanzania; 8 National Institute for Medical Research, Dodoma Research Centre, Benjamin Mkapa Hospital, Dodoma P.O. Box 805, Tanzania

**Keywords:** *Leptospira* antibodies, adults, febrile, Mwanza

## Abstract

Leptospirosis is a zoonotic neglected tropical disease with a worldwide distribution caused by the pathogenic spirochetes of the genus *Leptospira*. Despite being a widespread disease in tropical regions, it has never been considered in the routine diagnostic panel for febrile patients. This study determined seropositivity and factors associated with *Leptospira* antibodies among febrile adult patients in Mwanza, Tanzania. The cross-sectional study involving 296 febrile patients attending different outpatient clinics in Mwanza region was conducted between May and July 2019. Detection of *Leptospira* serovars antibodies was done using a microscopic agglutination test (MAT). Descriptive analysis was done using STATA version 13. The median age of the febrile patients was 32 (IQR: 24–45 years). Out of 296 patients, 36 (12.16%, 95%CI: 8–15) were seropositive for *Leptospira* antibodies. Common circulating serovars were Sokoine 28 (9.45%), followed by Lora 12 (4.05%) and Grippotyphosa 2 (0.67%). In the multivariable logistic regression analysis, the odds of being *Leptospira* seropositive were significantly higher with increased age (aOR: 1.03, 95%CI 1.00–1.07, *p* = 0.03). About one tenth of febrile patients in Mwanza were seropositive for *Leptospira* antibodies and this was significantly associated with age. With the decline of malaria fever in endemic areas, other causes of febrile illness like *Leptospiral* spp. should be considered in the routine diagnostic panel for febrile patients.

## 1. Introduction

*Leptospira* species which are etiological agents of Leptospirosis are gram negative bacteria belonging to the order Spirochaetales with over 230 serovars known to exist [1]. Leptospirosis is a febrile neglected tropical disease with a worldwide distribution and is associated with high morbidity and mortality [1,2]. The disease is zoonotic in nature, having both wild and domestic animals as natural reservoirs which include domestic livestock, peri domestic rats and mice, companion animals such as dogs, wild animals such as bats, marsupials and a variety of rodents [1]. Some serovars have specific reservoirs, such as *L. interrogans* serovar icterohaemorrhagiae, which are found primarily in rats [2,3]. 

Detecting antibodies of *Leptospira* serovars in the sera of reservoir animals residing in a particular region can represent the circulation among susceptible hosts in that region, emphasizing epidemiological studies in the occupational groups in contact with these animals. Transmission to humans occurs through exposure to the urine of the infected animals, either through direct contact or through contamination of water, soil, vegetation or handling of animals or their products [4].

*Leptospira* spp. Infection has an incubation period of 7–12 days with a range of 2–20 days and the diseases can present with fever, headache, severe myalgia, prostration, chills with rigor and sometimes circulatory collapse, which can be preceded by bleeding tendencies [5]. Leptospirosis affects millions with case fatality rates between 5% and 25% [6]. Reported median annual incidences is high in Africa, standing at 95.5 per 100,000 people, followed by Western Pacific (66.4), the Americas (12.5), Southeast Asia (4.8) and Europe (0.5) [7].

In Tanzania the disease is endemic, however there is a scarcity of information on its magnitude in different populations. A study done in Moshi, Northern Tanzania, reported 8.8% of confirmed leptospirosis cases among hospitalized patients, with Mini and Australis being predominant serogroups [8]. In Mwanza, 16.1% of tested samples from dog keepers had *Leptospira* antibodies, with *Leptospira* serovar Sokoine representing 94% of positive samples [9]. Among abattoir workers and meat sellers, 10% had *Leptospira* antibodies with 69.2% of positive samples being *Leptospira* serovar Sokoine [10]. This shows that *Leptospira* is common in Mwanza and might contribute to the cases of fever of unknown origin.

In recent years, malaria fever has experienced a downward trend in various areas of Tanzania necessitating the investigation of other causes of fever [11,12]. Despite the predominance of *Leptospira* antibodies among abattoir workers, meat sellers and dog keepers in Mwanza, its magnitude among febrile patients has not been established. The present study aimed at establishing baseline information on the magnitude of *Leptospira* spp. antibodies among febrile patients in Mwanza, Tanzania, information that might be useful in devising and sustaining control strategies. 

## 2. Materials and Methods

### 2.1. Study Design and Study Population

A cross-sectional, hospital-based study was conducted among febrile patients in different outpatient clinics in Mwanza region between May and July 2019. The blood samples were collected from Bugando Medical Centre (BMC) Outpatient Department (OPD), Sekou Toure Regional Referral Hospital (SRRH) Outpatient Department, Nyamagana District Hospital, Buzuruga Health Centre (BHC) and Sengerema Designated District Hospital (SDDH). In these health facilities, an average of 15 to 25 patients with fever were seen per day.

### 2.2. Sample Size Estimation, Sampling Procedures and Data Collection

Sample size was calculated by the Kish Leslie formula using the prevalence of 14.3% [13]. The minimum sample size was 188, however, a total of 296 patients were enrolled and included in the final analysis. Sociodemographic data was collected by using a pre-tested structured data collection tool. The tool included sociodemographic data such as age, sex, residency, marital status, employment status, economic status, level of education, medical history such as history of kidney disease, hematuria, diarrhea, vomiting etc., and clinical findings and information on other risk factors for leptospirosis. Blood samples were collected in plain vacutainer tubes (Becton Dickinson (E.A) Ltd., Nairobi, Kenya) from all consenting patients. Blood slides for detection of *Plasmodium* spp. and *Borrelia* spp. were prepared and examined under a light microscope. A blood culture was done for each sample to detect *Salmonella* spp. and other culturable blood stream bacterial pathogens as previously described [14]. Sera were extracted and stored in cryovials at −80 °C until processing. Detection of Chikungunya, Dengue and Zika virus were done by single-reaction multiplex real-time polymerase chain reaction (RT-PCR) as previously described [15]. All patients were PCR negative for Dengue, Chikungunya and Zika, smear negative for *Plasmodium* spp. and *Borrelia* spp. and culture negative for *Salmonella* spp. and other bacterial pathogens.

### 2.3. Laboratory Analysis of the Samples

Sera were transported to a pest management center at the Sokoine University of agriculture, Morogoro for laboratory analysis of *Leptospira* antibodies by using a microscopic agglutination test (MAT). MAT was performed as previously described [16]. Among the list of 10 *Leptospira* serovars recommended for leptospirosis diagnosis in Africa [7], five serovars, namely, *L. kirschneri* serovar Sokoine, *L. kirschneri* serovar Grippotyphosa, *L. interrogans* serovar Pomona, *L. interrogans* serovar Hebdomadis and *L. interrogans* serovar Lora, were selected (Table 1). The selected serovars represent the majority of serovars detected in Tanzania. Selected serovars were grown in a *Leptospira* Ellinghausen−McCullough−Johnson−Harris (EMHJ) medium containing 200µg/mL of 5-Fluorouracil as a selective inhibitor. The cultures were incubated for 4–7 days until a density of 3 × 10^8^ leptospires/mL was reached. Sera were then serially diluted from 1:10 to 1:80 and 50 µL of live antigen was added to double the dilution to 1:20 and 1:160. Thereafter, the mixtures were incubated at 30 °C for 2 h and examined for agglutination under dark field microscopy. Further titration was done for the samples reacting with a titer of 1:20 and above to determine the antibody levels and a cut-off point of ≥1:160 was considered positive [17]. To ensure quality, the tests were done simultaneously with both negative and positive controls.

### 2.4. Ethical Considerations

Ethical approval was sought from the joint CUHAS, BMC Research, Ethics and Review committee (CREC) and the study was given a clearance certificate number (CREC/1029/2019). Written informed consent was requested from each study participant prior to enrollment after receiving explanation on the importance of the study and related procedures. Patient confidentiality was maintained throughout the study.

### 2.5. Data Management and Analysis

Data were entered into an excel sheet for coding and cleaning and then transferred to STATA version 13 (Statistical Corporation, College Station, TX, USA) for analysis. Proportions were used to present categorical data such as sex, marital status, residency, occupation, having kidneys disease, history of hematuria etc., while median/interquartile range (IQR) was used to present continuous data such as age and household members. Bivariate analysis using a Chi square test was done. All factors with a *p* value equal to or less than 0.2 (age, residency, paddy cultivation, having rodents at home and joint pain) were subjected to multivariable logistic regression analyses; ninety-five percent confidence intervals (CI) and adjusted odds ratios were recorded to show the strength of the association between *Leptospira* seropositivity and independent variables. A *p* value of <0.05 at 95% confidence interval was considered statistically significant.

## 3. Results

### 3.1. Sociodemographic Characteristics of the Enrolled Febrile Adults (n = 296)

A total of 296 febrile adult patients were included in the study with a median age of 32 (IQR: 24–45 years). The median number of household members was 6 (IQR: 4–7 family members). More than three-quarters (226, 76.35%) of patients were females and the majority 237(80.07%) used modern toilets. Most of the participants (248, 83.78%) used tap water as their source of water and about one third (100, 33.78%) owned small businesses. More than three-quarters (226, 76.5%) resided in urban areas of Mwanza and the majority (257, 86.82%) had brick/iron houses. More than half (157, 53.58%) attained a secondary education and the majority (213, 71.96%) were married (Table 2).

### 3.2. Clinical Characteristics of Enrolled 296 Febrile Adults

Regarding clinical characteristics, the majority (177, 60.82%) of patients had no joint pain, myalgia (175, 60.55%), hematuria (212, 98.60%) or bloody diarrhea (205, 99.51%) (Table 3).

### 3.3. Seropositivity of Leptospira Antibodies among Adult Febrile Patients in Mwanza

Out of 296 adult febrile patients tested, 36(12.16%, 95%CI: 8–15) were seropositive for *Leptospira* antibodies. According to two sample Wilcoxon rank-sum (Mann-Whitney) tests, the median age of *Leptospira* seropositive patients was significantly higher than that of their seronegative counterparts (40 IQR 30–56 vs. 32 IQR 24–43.50, *p* < 0.001).

### 3.4. Factors Associated with Leptospira Seropositivity among 296 Febrile Adults in Mwanza Region

In the univariate regression analysis, being *Leptospira* seropositive was significantly associated with an increase in age (*p* = 0.007). In the multivariable logistic regression analysis, only advanced age (OR: 1.03, 95%CI: 1.01–1.06, *p* = 0.007) was significantly associated with *Leptospira* spp. seropositivity among febrile patients in Mwanza (Table 4).

### 3.5. Circulating Leptospira Serovars among Febrile Adults in Mwanza Region

The sera from 296 febrile adults were tested for antibodies against five *Leptospira* serovars. Five (1.69%) febrile adults were seropositive to more than one serovar. Of the 36 *Leptospira* seropositive results, serovar Grippotyphosa, Lora and Sokoine accounted for 2(0.67%), 12(4.05%) and 28(9.45%) seropositive results, respectively.

## 4. Discussion

To the best of our knowledge, this is the first report to document the seropositivity of *Leptospira* spp. among febrile patients in Mwanza, Tanzania. The previous reports in the same settings documented *Leptospira* seropositivity among abattoir workers, meat vendors and dog keepers [9,10].

The seropositivity of *Leptospira* spp. antibodies in the current study was found to be 12.2%, which is comparable with a previous study in Pondicherry, India that reported the positivity of 12% [5]. This could be explained by both studies having similar study participant characteristics. No significant differences were observed regarding the seropositivity reported in the current study compared to a previous study among abattoir workers and meat vendors in the same setting, which reported a seropositivity of 10% [10].

In comparison to a previous study among dog keepers in Mwanza which reported a seropositivity of 16.1%, the reported seropositivity in the current study is slightly low [9]. This may be explained by the fact that the previous study enrolled a group at a higher risk for *Leptospira* than those included in the current study. When compared to a previous report in Katavi that reported a seropositivity of 29.9%, the seropositivity in this study is significantly lower [18]. This could be explained by the fact that in the current report, only a small number of serovars were investigated compared to the previous study.

Among the factors studied, only advanced age was found to be an independent predictor for *Leptospira* spp. Seropositivity, which is different from previous studies in Mwanza, Germany and Mexico [2,10,15]. Like many other diseases in endemic areas, having advanced age has been associated with increased exposure to the risk factors when compared with those of a young age. Despite having a non-significant association, the seropositivity of *Leptospira* antibodies was slightly high in males compared to their counterparts, and married individuals also appeared to have slightly higher seropositivity than unmarried ones. As previously reported in the same settings, the interaction with animals is more in males than in females in the study area [10]. Further studies to investigate the role of sex and marital status in relation to *Leptospira* seropositivity are warranted in the study area.

As previously reported in the same settings, among the serovars tested in the current study, *L. kirschneri* serovar Sokoine was found to be predominant (9.5%) compared to other serovars [9,10]. This shows that *L. kirschneri* serovar Sokoine is the most common serovar circulating in Mwanza. Considering its discovery in cattle from slaughterhouses in Morogoro, Tanzania more than 15 years ago [19], there is a paramount need to investigate its magnitude among domesticated animals including cattle and other human populations in Tanzania and other similar settings in low- and middle-income countries. Due to its zoonotic nature, this observation calls for One Health approach strategies in efforts to control leptospirosis in endemic areas. This observation is different from a previous study in Germany which documented *L. interrogans* serovar Grippotyphosa as the most common serovar (9%) [2] while in the current study, this serovar is the least common. This can be explained by the lack of reservoirs, such as field moles and European hamsters, in Mwanza, which were responsible for the transmission of the serovar in Germany [2].

### Study Limitations

The seropositivity of *Leptospira* antibodies in the current study might be underestimated due to the fact that, among the 10 serovars recommended in the diagnosis of Leptospirosis in Africa by using MAT, only five were selected and included in the panel. Nevertheless, the five selected serovars used in the current study formed the majority in previous studies that used a complete panel.

## 5. Conclusions

Twelve percent of febrile adults in Mwanza have *Leptospira* antibodies, which is significantly associated with advanced age. With increased human−animal interactions in Tanzania and other LMICs, there is a need for continuous surveillance in areas with high interactions so that the outbreaks can be detected early to prevent associated morbidity and mortality. Furthermore, this calls for the need to consider *Leptospira* spp. in the routine workup of patients with a fever in endemic areas and the One Health approach for its control.

## Figures and Tables

**Table 1:** Species, serogroups, serovars and strains used in MAT.

Serial Number	Species	Serogroups	Serovars	Strains
1	*L. kirschneri*	Icterohaemorrhagiae	Sokoine	RMI-Cattle
2	*L. kirschneri*	Grippotyphosa	Grippotyphosa	Moskva-V
3	*L. interrogans*	Pomona	Pomona	Pomona
4	*L. interrogans*	Hebdomadis	Hebdomadis	Hebdomadis
5	*L. interrogans*	Australis	Lora	TE1992

**Table 2:** Sociodemographic characteristics of 296 febrile adults in Mwanza, Tanzania.

Variable		Number (n)/Median/Mean	Percentage (%)/IQR/SD
Age (years)		32	24–45
People in household		6	4–7
Sex	Female	226	76.35
	Male	70	23.65
Residence	Rural	70	23.65
	Urban	226	76.35
Water source	Lake or pond	48	16.22
	Tap water	248	83.78
Toilet	Modern	237	80
	Pit latrine	59	19.93
House type	Brick/iron	257	86.82
	Mud/mud	39	13.18
Education	Primary	114	38.91
	Secondary	157	53.58
	University/College	22	7.51
Marital status	Married	213	71.96
	Single	83	28.04
Occupation	Business	100	33.78
	Employed	45	15.20
	Housewife	103	34.80
	Student	48	16.22

**Table 3:** Clinical characteristics of enrolled febrile adults in Mwanza.

Variable		Number	Percentage (%)
Hematuria	No	212	98.60
	Yes	3	1.40
Micturia	No	180	83.72
	Yes	35	16.28
Bloody diarrhea	No	205	99.51
	Yes	1	0.49
Joint pain	No	177	60.82
	Yes	114	39.18
Headache	No	33	11.15
	Yes	263	88.85
Myalgia	No	175	60.55
	Yes	114	39.45

**Table 4:** Univariate and multivariate logistic regression analysis of the factors associated with *Leptospira* seropositivity among 296 febrile adults in Mwanza, Tanzania.

Overall Leptospirosis			Univariate Analysis		Multivariable Analysis	
Characteristics		Seropositivity (%)	Chi-Square/Odds Ratio (95%CI)	*p*-Value	Odd’s Ratio (95%CI)	*p*-Value
Age (years)		** 40(IQR 30–56)	* 1.02(1.01–1.04)	0.007	1.03(1.01–1.06)	0.007
Sex	Female (226)	25(11.06)				
	Male (70)	11(15.71)	1.08	0.30		
Residence	Urban (226)	24(10.62)				
	Rural (70)	12(17.14)	2.13	0.14	1.22(0.49–2.99)	0.77
Education	Primary (114)	13(11.40)				
	Tertiary (22)	1(4.55)				
	Secondary (157)	22(14.01)	1.74	0.28		
Household (people)		** 6(IQR 4–7)		0.91		
Marital status	Single (83)	7(8.43)				
	Married (213)	29(13.62)	1.74	0.22		
Paddy cultivation	No (155)	12(7.74)				
	Yes (32)	10(12.50)	1.41	0.11	0.35(0.11–1.02)	0.06
Sewage	No (60)	9(15.00)				
	Yes (236)	27(11.44)	0.57	0.45		
Rodent at home	No (142)	12(8.45)				
	Yes (73)	8(10.96)	0.36	0.06	0.57(0.25–1.29)	0.18
Joint pain	No (177)	18(10.17)				
	Yes (114)	18(15.79)	2.02	0.13	1.49(0.68–3.2)	0.312
Headache	No (33)	5(15.15)				
	Yes (263)	31(11.79)	0.31	0.58		
Myalgia	No (175)	19(10.86)				
	Yes (114)	17(14.91)	1.04	0.25		
Conjunctivitis	No (189)	16(8.47)				
	Yes (17)	3(17.65)	1.57	0.48		

* logistic regression has been done, ** median and interquartile range.

## Data Availability

All information has been included in the manuscript.
